# Selective bird predation on the peppered moth: the last experiment of Michael Majerus

**DOI:** 10.1098/rsbl.2011.1136

**Published:** 2012-02-08

**Authors:** L. M. Cook, B. S. Grant, I. J. Saccheri, J. Mallet

**Affiliations:** 1 Life Sciences, University of Manchester, Manchester M13 9PT, UK; 2 Department of Biology, College of William and Mary, Williamsburg, VA 23187, USA; 3 Institute of Integrative Biology, University of Liverpool, Liverpool L69 7ZB, UK; 4 Genetics Evolution and Environment, UCL, 4 Stephenson Way, London NW1 2HE, UK; 5 Department of Organismic and Evolutionary Biology, Harvard University, 26 Oxford Street, Cambridge, MA 02138, USA

**Keywords:** natural selection, cryptic coloration, ecological genetics, insectivorous birds, melanism, lepidoptera

## Abstract

Colour variation in the peppered moth *Biston betularia* was long accepted to be under strong natural selection. Melanics were believed to be fitter than pale morphs because of lower predation at daytime resting sites on dark, sooty bark. Melanics became common during the industrial revolution, but since 1970 there has been a rapid reversal, assumed to have been caused by predators selecting against melanics resting on today's less sooty bark. Recently, these classical explanations of melanism were attacked, and there has been general scepticism about birds as selective agents. Experiments and observations were accordingly carried out by Michael Majerus to address perceived weaknesses of earlier work. Unfortunately, he did not live to publish the results, which are analysed and presented here by the authors. Majerus released 4864 moths in his six-year experiment, the largest ever attempted for any similar study. There was strong differential bird predation against melanic peppered moths. Daily selection against melanics (*s* ≃ 0.1) was sufficient in magnitude and direction to explain the recent rapid decline of melanism in post-industrial Britain. These data provide the most direct evidence yet to implicate camouflage and bird predation as the overriding explanation for the rise and fall of melanism in moths.

## 1. Introduction

Melanism in the peppered moth *Biston betularia* led to the earliest measurements of natural selection on a Mendelian locus in the wild [1,2]. Rapid nineteenth century increases in melanics, followed by more recent declines took place in step with changing patterns of industrialization in Britain and elsewhere [3–5]. The melanic ‘*carbonaria*’ morph is inherited via a dominant allele, *C*, at a single locus. The recessive *c* allele specifies the non-melanic black and white ‘*typica*’ form, while intermediate melanic ‘*insularia*’ alleles, inherited at the same locus, are dominant to *typica* and recessive to *carbonaria. Insularia* forms also increased, somewhat variably, during industrialization [5]. Recently, this locus has been mapped and cloned; the pattern of genetic variation in the genomic region harbouring the *C* locus suggests a rapid selective sweep around a single mutational origin of melanism [6].

Melanics were long believed to be advantageous in the face of bird predation against bark resting sites darkened by soot pollution [2], a form of camouflage [7]. Classic experiments in the mid-twentieth century proved that birds attacked the moths. Furthermore, resting moths that failed to match their background were more vulnerable to bird predation in cage experiments [8,9]. Mark–recapture studies of live moths, as well as many bird predation experiments using dead moths pinned to tree trunks, supported the hypothesis that birds were the agents of selection on melanism [3,9].

However, these procedures have drawbacks [3,5,10], and critiques were increasingly aired [5,11,12]. In experiments, moths were often placed on tree trunks, which were argued to be abnormal resting sites; pinned carcases seemed particularly unnatural. Moths were often released at greatly inflated densities, potentially increasing predation. Reared insects from geographically distant sources were often used to supplement wild individuals, and may not have behaved as naturally in recapture experiments as wild moths. By the 1990s, considerable scepticism became evident [11–14]. Factors other than bird predation (e.g. migration, physiological differences among genotypes) were argued to play a substantial role in the evolution of melanism in *Biston* [5,15–17]. Caveats about the predation experiments discussed in Majerus's book [5], critiques by other biologists, as well as points made particularly forcefully in a review of the Majerus book [18], were soon exploited by non-scientists to promote an anti-evolution agenda and to denigrate the predation explanation [10]. Kettlewell's original mark–recapture experiments were later argued to be fraudulent [19] (quite groundlessly: see [3,10,20]). Judith Hooper, author of this claim, also suggested that bats rather than birds might be the agents of selection [19]. Soon, both the public in general and even evolutionary biologists began to doubt the bird predation story (electronic supplementary material, S1 [18]).

Majerus therefore decided to make key new field observations, and he also designed and carried out a massive new predation experiment, the largest predation experiment ever performed (4864 released moths) to answer his own and other criticisms of earlier work [5,10,21]. In addition, to address the possible effect of bat predation, Majerus released live moths in night-time experiments in multiple locations: he found no significant differences in predation of 419 melanic and typical moths eaten by three species of pipistrelle bats [22].

These results were all presented by Majerus in a keynote address at the ESEB Congress, Uppsala, in August 2007. Unfortunately, Majerus died after a short illness in 2009 before publishing the resting site observations and predation results. However, the information from the Uppsala talk, which forms the basis for the current analysis, was made freely available by Majerus on the Internet soon thereafter as a set of projected slides. We have formed the current collaboration in order to analyse these results and disseminate them in print for the first time, as well as to clarify the importance of these key results for our understanding of natural selection in the wild.

## 2. Material and methods

Experimental and observational work presented here was carried out by Michael Majerus in a 1 ha rural garden, at Springfield, near Coton, Cambridgeshire, UK. Full methods were published in a little-known chapter [10]; relevant extracts are provided here (electronic supplementary material, S2). Original source files of his presentation in 2007, which contain the results analysed here, are provided at http://dx.doi.org/10.5061/dryad.962262h9. In cases where numerical data were not supplied in these files, we have expressed the results in terms similar to those used by Majerus in the 2007 documents.

### (a) Natural resting sites

While climbing trees in the experimental site in order to set up sleeves for the predation experiment (see below), Majerus systematically scrutinized trunks, branches and twigs of a limited set of trees and recorded natural resting positions of all wild moths he found. The 135 observations he obtained here add considerably to the less-extensive resting site data previously published [10,12].

### (b) Predation experiment

The purposes of the new experiment by Majerus were to estimate the relative survival of local melanic and non-melanic moths at low, naturalistic densities. Moths (melanic *carbonaria* and non-melanic *typica*; no *insularia* morphs were used) were therefore released on substrates that Majerus himself had shown were normal for the species. Morphs were released at frequencies close to those estimated from captures in the previous year at Madingley, near Cambridge. (These Madingley frequencies have been interpolated by us from a graph provided by Majerus in his 2007 presentation; see table 2, column 2).

A total of 4864 peppered moths were released during the natural emergence seasons over 6 years. Each night one moth was released into each of the 12 netting ‘sleeves’ surrounding a branch selected randomly from among the 103 (reduced to 97 by 2007) branches used in the study. Sleeves and any moths resting on them were removed before dawn, and positions of moths remaining undisturbed on bark were noted. Release density averaged less than 10 moths ha^−1^ night^−1^. Moths absent from resting positions 4 h after sunrise were presumed eaten by predators as they rarely fly away during daylight unless greatly disturbed. Of those that disappeared, approximately 26 per cent were seen being eaten by birds via binocular observations [10]. For further details, see electronic supplementary material, S2 and the study of Majerus [10].

### (c) Statistical analysis of predation experiment

Data from the predation experiment (table 2, columns 4–6) were provided as a three-way contingency table. The numbers of *carbonaria* and *typica* released yearly were fixed by the experimental design. Such non-standard contingency tables are readily handled by log-linear models [23]. Maximum-likelihood values of parameters of non-selective and selective predation rates were estimated given the assumptions of homogeneity or heterogeneity of effects among years, and likelihood-ratio tests were performed to test models of different hierarchical complexity [24,25]. While simple contingency table analysis is probably more familiar, non-selective predation was heterogeneous among years, and a more complex analysis that takes account of the heterogeneity was required (see electronic supplementary material, S3).

## 3. Results

### (a) Natural resting sites

Majerus had already shown that some moths did normally rest on tree trunks during the day [10]. His recent extensive observations on resting sites obtained while climbing the trees in his garden to set up the predation experiment appear to have been lost, but he summarized the work in his 2007 Uppsala presentation (http://dx.doi.org/10.5061/dryad.962262h9). An annotated version of this summary is presented here. Majerus reported the following major features (table 1):

— The majority (52%) of moths rest on lateral branches.
— Of the moths on lateral branches, the majority (89%) rest on the lower half of the branch.
— A significant proportion of moths (35%) rest on tree trunks.
— Of those that rest on trunks, the majority (87%) rest on the north, rather than the south half.
— A minority of moths (13%) rest under or among twigs.
— There were no significant differences in the resting sites of males and females (*χ*^2^ ≈ −2*Δ*ln *L* = 0.33, 2 d.f.).
— There were no significant differences in the resting sites used by *typica* (non-melanic), *carbonaria* (full melanic) or *insularia* (intermediate melanic) forms (numerical data not available).

**Table 1.** Numbers of wild peppered moths observed in different daytime resting positions, 2001–2006. Previous authors had argued that moths rarely rested on tree trunks during the day, and that many predation experiments employing tree trunks were therefore unnatural. In these new observations by Majerus, 35% of the 135 moths observed, both melanic and typical, were indeed found resting on tree trunks.

	trunks	branches	twigs	total
males	28	40	11	79
females	20	30	6	56
totals	48	70	17	135

The findings about orientation of resting position suggest avoidance of exposure to sun, while also verifying that a reasonably high fraction of moths do rest on the trunks of trees, as well as in the canopy.

Note that these observations were of wild moths which did not form part of the predation experiment.

### (b) Predation experiment

**Figure 1.**
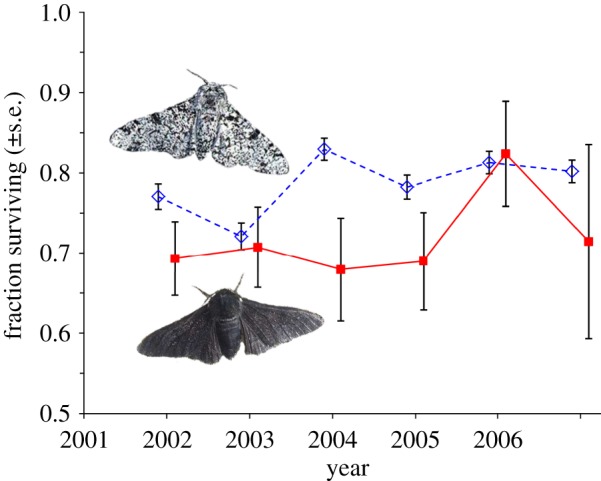
Survival of moths (±s.e.) over the course of the predation experiment. Unfilled diamonds with dashed lines, non-melanic; filled squares with solid lines, melanic.

In Majerus's new predation experiment at an unpolluted site, significantly more released melanics than non-melanics disappeared, or were seen to be eaten by nine bird species (figure 1 and table 2). This lower fitness of melanics is expected if the observed decline in melanism (table 2) is explained by visual predation.

**Table 2.** Survival of moths in the predation experiment in different years. Column 2: Frequency of wild melanics (*carbonaria*) obtained in light trap samples at Madingley, near Cambridge. Columns 3–6: Typical and melanic (*carbonaria*) individuals made available and eaten at the experimental site in suburban Cambridge, UK. Expected values under null (*e* ∼*s*) and best-fit (heterogeneous overall survival and homogeneous predator selection among years, *eee s*) models are shown, respectively, in parentheses. Nine bird species were observed eating the moths: English robins (*Erithacus rubecula*), hedge sparrows (*Prunella modularis*), great tits (*Parus major*), blue tits (*Cyanistes caeruleus*), European blackbirds (*Turdus merula*), starlings (*Sturnus vulgaris*), Eurasian wrens (*Troglodytes troglodytes*), magpies (*Pica pica*) and a lesser-spotted woodpecker (*Dendrocopus minor*).

year	local melanic frequency	typicals available	melanics available	typicals eaten	melanics eaten
2001	0.12				
2002	0.10	706	101	162 (154.29, 162.52)	31 (22.07, 30.32)
2003	0.06	731	82	204 (159.76, 200.83)	24 (17.92, 27.94)
2004	0.07	751	53	128 (164.13, 130.65)	17 (11.58, 13.20)
2005	0.04	763	58	166 (166.75, 166.90)	18 (12.68, 16.81)
2006	0.02	774	34	145 (169.15, 143.00)	6 (7.43, 8.80)
2007	0.01	797	14	158 (174.18, 158.15)	4 (3.06, 3.80)

The full statistical analysis (electronic supplementary material, S3) demonstrates:

— strong evidence of heterogeneity in the non-selective fraction eaten of both morphs across years (*p* < 0.001), but
— no significant variation in the fraction selectively eaten across years, and
— strong evidence of overall selection against the melanic form (*p* = 0.003), while taking into account the year-to-year heterogeneity in the non-selective fraction eaten.
— The maximum likelihood selective coefficient against melanics is *s* = 0.091 per day (with likelihood-estimated 95% confidence intervals of 0.028–0.157). This gives daily relative survival estimates for melanics of 91 per cent (84–97%) of that of the typical forms. These estimates and confidence intervals account for year-to-year heterogeneity in overall survival (first result, above).

## 4. Discussion

The lifespan of wild moths is several days, so the approximately 9 per cent reduction in daily survival of melanics is sufficient in magnitude and direction to explain their long-term local decline; the decline rate suggests a selection pressure against melanics of *s* ≈ 0.1–0.2 per generation (table 2; [3]). Majerus was able to see predation events from his window, involving nine species of local insectivorous birds (table 2). Clearly melanics disappeared faster than non-melanics in this experiment, and Majerus was able to confirm by direct observation that about one-quarter of the disappearances were owing to bird predation [10].

Factors other than predation have often been argued to play a substantial role in the rise and subsequent post-industrial fall of melanism in *Biston* [5,15–17]. Nonetheless, with this new evidence added to the existing data, it is virtually impossible to escape the previously accepted conclusion that visual predation by birds is the major cause of rapid changes in frequency of melanic peppered moths [3,5]. These new data answer criticisms of earlier work and validate the methodology employed in many previous predation experiments that used tree trunks as resting sites [3]. The new data, coupled with the weight of previously existing data convincingly show that ‘industrial melanism in the peppered moth is still one of the clearest and most easily understood examples of Darwinian evolution in action’ [21].
